# Diagnostic accuracy of a novel point-of-care urine lipoarabinomannan assay for the detection of tuberculosis among adult outpatients in Zambia: a prospective cross-sectional study

**DOI:** 10.1183/13993003.03999-2020

**Published:** 2021-11-18

**Authors:** Monde Muyoyeta, Andrew D. Kerkhoff, Lophina Chilukutu, Emmanuel Moreau, Samuel G. Schumacher, Morten Ruhwald

**Affiliations:** 1 Centre for Infectious Diseases Research in Zambia, Lusaka, Zambia; 2 Division of HIV, Infectious Diseases and Global Medicine, Zuckerberg San Francisco General Hospital and Trauma Center, University of California San Francisco, San Francisco, CA, USA; 3 Foundation for Innovative New Diagnostics (FIND), Geneva, Switzerland; 4 These authors contributed equally to this work

## Abstract

**Background:**

A novel, rapid, point-of-care urine-based lipoarabinomannan assay (Fujifilm SILVAMP TB LAM (“FujiLAM”)) has previously demonstrated substantially higher sensitivity for tuberculosis (TB) compared with the commercially available Determine TB LAM assay using biobanked specimens. However, FujiLAM has not been prospectively evaluated using fresh urine specimens. Therefore, we determined the diagnostic accuracy of FujiLAM among HIV-positive and HIV-negative outpatients with presumptive TB in Zambia.

**Methods:**

Adult (≥18 years old) presumptive TB patients presenting to two outpatient public health facilities in Lusaka were included. All patients submitted sputa samples for smear microscopy, Xpert MTB/RIF and mycobacterial culture, and urine samples for the FujiLAM assay. Microbiologically confirmed TB was defined by the detection of *Mycobacterium tuberculosis* in sputum using culture; this served as the reference standard to assess the diagnostic accuracy of FujiLAM.

**Results:**

151 adults with paired sputum microbiological tests and urine FujiLAM results were included; 45% were HIV-positive. Overall, 34 out of 151 (23%) patients had culture-confirmed pulmonary TB. The overall sensitivity and specificity of FujiLAM was 77% (95% CI 59–89%) and 92% (95% CI 86–96%), respectively. FujiLAM's sensitivity among HIV-positive patients was 75% (95% CI 43–95%) compared with 75% (95% CI 51–91%) among HIV-negative patients. The sensitivity of FujiLAM in patients with smear-positive, confirmed pulmonary TB was 87% (95% CI 60–98%) compared with 68% (95% CI 43–87%) among patients with smear-negative, confirmed pulmonary TB.

**Conclusions:**

FujiLAM demonstrated high sensitivity for the detection of TB among both HIV-positive and HIV-negative adults, and also demonstrated good specificity despite the lack of systematic extrapulmonary sampling to inform a comprehensive microbiological reference standard.

## Abstract 2

**In a prospective study, the FujiLAM urine-based point-of-care test demonstrated high sensitivity of ≥75% for the detection of TB among both HIV-positive and HIV-negative ambulatory adults, and also had good specificity.** https://bit.ly/328jr0d

## Introduction

The development of new, highly sensitive, point-of-care tuberculosis (TB) diagnostic tests that are not dependent on a sputum sample are key to meeting the End TB Strategy targets [1]. These diagnostic tests should be affordable and easy to use, to facilitate scale-up in high-burden, resource-constrained settings where there is the most need. In 2019, the World Health Organization (WHO) strengthened and expanded upon its recommendations on the use of the Determine TB LAM (“LF-LAM”) test (Abbott Diagnostics, Lake Bluff, IL, USA), a rapid, urine-based, point-of-care assay, to diagnose TB among people living with HIV (PLHIV) [2].

The LF-LAM test is based on the detection of mycobacterial lipoarabinomannan (LAM). LAM is a cell wall byproduct of replicating mycobacterial bacilli that can be detected in the blood, sputum and urine of persons with TB, although development of urine-based LAM assays is attractive due to ease of specimen collection. LAM is a heterogenous molecule with four structural domains, including a domain highly preserved across mycobacterial species as well as variable domains that may serve as target epitopes for the development of TB diagnostic tests [3, 4]. Despite LF-LAM's low cost, ease of use and demonstrated mortality benefit, its uptake has been slow [5]. This is largely due to its low sensitivity in most patient groups, with the exception being individuals with advanced immune deficiency [6].

The Fujifilm SILVAMP TB LAM (“FujiLAM”) (Fujifilm, Tokyo, Japan) is a next-generation, rapid, urine-based LAM test that has been demonstrated to have two-fold higher sensitivity than LF-LAM in PLHIV (71% *versus* 35%) using biobanked specimens and it also retained useful sensitivity at higher CD4 count levels as well as among HIV-negative individuals [7, 8]. Compared with LF-LAM, which is a lateral flow assay that uses polyclonal antibodies, FujiLAM utilises high-affinity monoclonal antibodies directed towards largely *M. tuberculosis*-specific LAM epitopes and adds a silver amplification step; this facilitates detection of LAM concentrations 30 times lower than can be detected using the LF-LAM assay, while also providing improved analytic specificity [9]. However, the diagnostic accuracy of the FujiLAM assay has not previously been prospectively evaluated on fresh urine specimens and to date the available data among people without HIV are very limited [8]. We report and compare the diagnostic accuracy of FujiLAM among HIV-positive and HIV-negative presumptive TB patients in an outpatient setting, using fresh specimens.

## Methods

### Study design and participants

This diagnostic evaluation study was nested within a larger, ongoing TB diagnostic study. Adults (≥18 years old) attending Kanyama First Level Hospital and Chainda Health Centre (Lusaka, Zambia) between January 2019 and July 2019 were screened for the presence of any TB symptoms. Kanyama First Level Hospital serves a large densely populated peri-urban township with a high prevalence of HIV and incidence of TB; TB notification rates typically exceed >2000 per 100 000 in this setting. Adult presumptive TB patients without a current TB diagnosis were eligible for study inclusion and those who agreed to participate were consecutively enrolled. Patients were defined as having “presumptive TB” if any of the following symptoms were present (regardless of duration): cough, fever, night sweats or unintentional weight loss, or abnormal chest radiography (regardless of symptoms).

The study had ethical approval from the Biomedical Research Ethics Committee of the University of Zambia (Lusaka, Zambia). All participants provided written informed consent in their primary language.

### Procedures and samples

All patients completed a case record form that collected demographic details, presenting symptoms, past medical history, HIV status and physical examination findings. HIV status was by self-report; however, for those without a known or recently determined HIV status, they were offered opt-out HIV testing in accordance with local procedures. Participants were offered digital chest radiography (Odelca-DR; Delft Imaging Systems, ’s-Hertogenbosch, The Netherlands) when available as part of the screening process. All patients submitted sputum and urine samples that were sent to an accredited laboratory for same-day processing. Sputum samples were processed for mycobacterial culture using the NALC-NaOH method: the pellet was inoculated on mycobacteria growth indicator tubes (MGIT; Becton Dickinson, Franklin Lakes, NJ, USA) and Löwenstein–Jensen medium (Becton Dickinson). The leftover pellet was used for Xpert MTB/RIF testing and concentrated acid-fast bacilli (AFB) Ziehl–Neelsen testing. Culture-positive tubes confirmed to contain AFB were tested with the MGIT TBc identification test (Becton Dickinson) to confirm the presence of *M. tuberculosis* [10]. The FujiLAM assay was performed on fresh urine specimens on the same day as sample collection according to the manufacturer's instructions [11]. CD4 cell counts were not systematically performed as part of this study.

### Data analysis and definitions

Patient characteristics were compared according to HIV status using simple descriptive statistics. Proportions were compared using either Pearson's Chi-squared test or Fisher's exact test as appropriate, while the Wilcoxon rank-sum test was used to compare median values. The detection of *M. tuberculosis* in sputum using culture defined the microbiological reference standard for diagnostic accuracy calculations. The sensitivity, specificity, positive predictive value (PPV) and negative predictive value (NPV) with corresponding 95% confidence intervals were determined for sputum smear microscopy, Xpert MTB/RIF and FujiLAM. The sensitivities of microbiological TB investigations for culture-confirmed TB were compared and visualised using proportional Venn diagrams. To better understand the association between FujiLAM positivity and mycobacterial burden, we determined the sensitivity of FujiLAM classified according to sputum Xpert MTB/RIF semiquantitative result (*e.g.* very low, low, medium or high).

## Results

**FIGURE 1:**
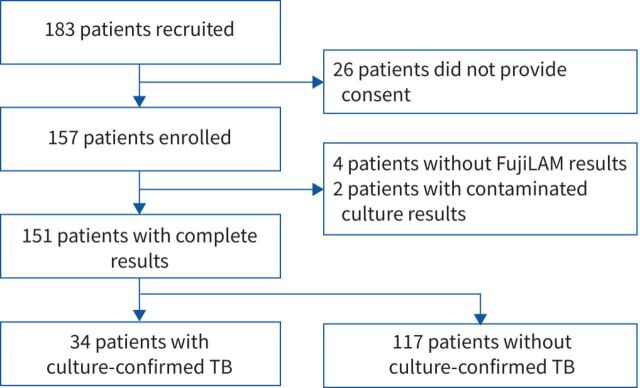
Study flow diagram. TB: tuberculosis.

Of 183 adult presumptive TB patients recruited, 157 were enrolled; 151 had complete microbiological results and were included in the analysis (figure 1). 89 (59%) patients were male, the median (interquartile range) age was 37 (28–43) years, 68 (46%) were HIV-positive and 32 (21%) had a prior history of TB. Compared with HIV-negative patients, HIV-positive patients were slightly older, less likely to report cough or fevers and were substantially more likely to report a prior history of active TB disease (table 1). Overall, 34 out of 151 (23% (95% CI 16–30%)) patients had culture-confirmed pulmonary TB; the prevalence of TB was 18% (95% CI 9–29%) among HIV-positive patients and 25% (95% CI 16–36%) among HIV-negative patients.

**TABLE 1:** Patient characteristics according to HIV status

	**All patients** **^#^**	**HIV-positive patients**	**HIV-negative patients**
**Patients**	151	68	81
**Age years**	37 (28–43)	39 (31–44)	33 (26–43)
**Male**	89 (58.9)	37 (54.4)	50 (61.7)
**Symptoms (any duration)**			
Cough	123 (81.5)	51 (75.0)	70 (86.4)
Fevers	81 (53.6)	29 (42.7)	50 (61.7)
Weight loss	109 (72.2)	49 (72.1)	58 (71.6)
Night sweats	106 (70.2)	44 (64.7)	61 (75.3)
Haemoptysis	22 (14.6)	10 (14.7)	11 (13.6)
**WHO symptom screen-positive**	145 (96.0)	63 (92.7)	80 (98.8)
**New HIV diagnosis**	2 (1.3)	2 (2.9)	
**Previous history of TB**	32 (21.2)	27 (39.7)	5 (6.2)
**Sputum culture TB-positive**	34 (22.5)	12 (17.7)	20 (24.7)

Data are presented as n, median (interquartile range) or n (%). WHO: World Health Organization; TB: tuberculosis. ^#^: two patients did not have HIV status results available.

### Diagnostic accuracy of FujiLAM

**FIGURE 2:**
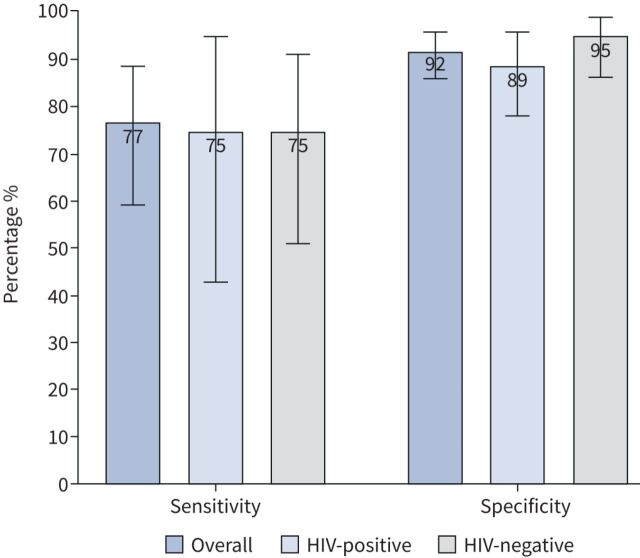
Diagnostic sensitivity and specificity of FujiLAM overall (n=151 patients) and according to HIV status (HIV-positive n=68 patients and HIV-negative n=81 patients). Two patients did not have HIV status results available. Error bars indicate 95% CI.

FujiLAM had an overall sensitivity and specificity of 77% (95% CI 59–89%) and 92% (95% CI 86–96%), respectively (figure 2). Among HIV-positive individuals, the sensitivity and specificity of FujiLAM was 75% (95% CI 43–95%) and 89% (95% CI 78–96%), respectively, while among HIV-negative individuals the sensitivity and specificity was 75% (95% CI 51–91%) and 95% (95% CI 86–99%), respectively. FujiLAM's sensitivity among those with smear-positive TB was 87% (95% CI 60–98%); among smear-negative individuals, the sensitivity and specificity of FujiLAM was 68% (95% CI 43–87%) and 92% (95% CI 86–96%), respectively (supplementary table S1). The characteristics of the nine patients with a “false-positive” FujiLAM result are shown in supplementary table S2; six patients were HIV-positive, three had a prior history of TB, eight had current cough, six had recent weight loss, five had current night sweats and four had current fever; one patient had a positive Xpert MTB/RIF result.

The overall PPV for FujiLAM was 74% (95% CI 57–88%); PPVs among HIV-infected and HIV-negative patients were 60% (95% CI 32–84%) and 83% (95% CI 59–96%), respectively (supplementary table S3). The overall NPV for FujiLAM was 93% (95% CI 87–97%); NPVs among HIV-infected and HIV-negative patients were 94% (95% CI 84–99%) and 92% (95% CI 82–97%), respectively.

### Comparative diagnostic sensitivity of tuberculosis tests

**FIGURE 3:**
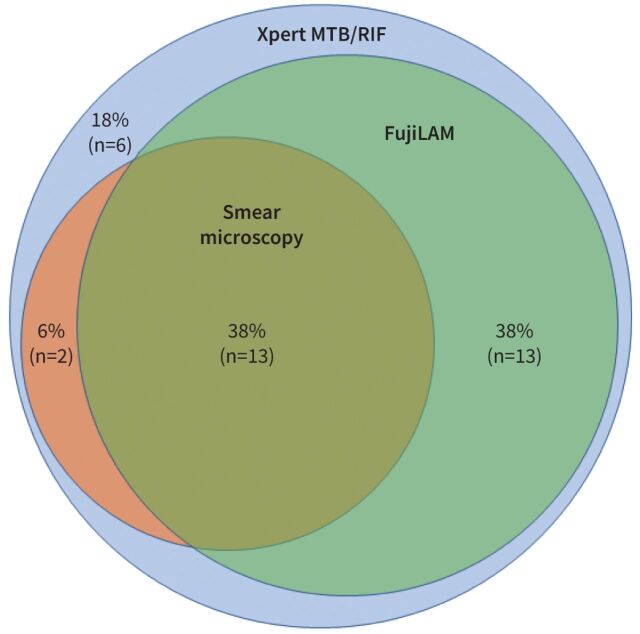
Proportional Venn diagram showing the diagnostic sensitivity and number of culture-confirmed tuberculosis cases detected using different assays (n=34 patients).

The comparative diagnostic sensitivity of different TB tests among those with culture-confirmed, pulmonary TB is shown in figure 3. FujiLAM demonstrated higher diagnostic sensitivity compared with sputum smear microscopy (77% *versus* 44%) (supplementary table S1). While the addition of FujiLAM to sputum smear microscopy increased sensitivity by 38% (n=13 cases), the additive sensitivity of sputum smear microscopy following FujiLAM was only 6% (n=2 cases). All TB cases were detectable using Xpert MTB/RIF.

### Relationship between FujiLAM and mycobacterial burden

**FIGURE 4:**
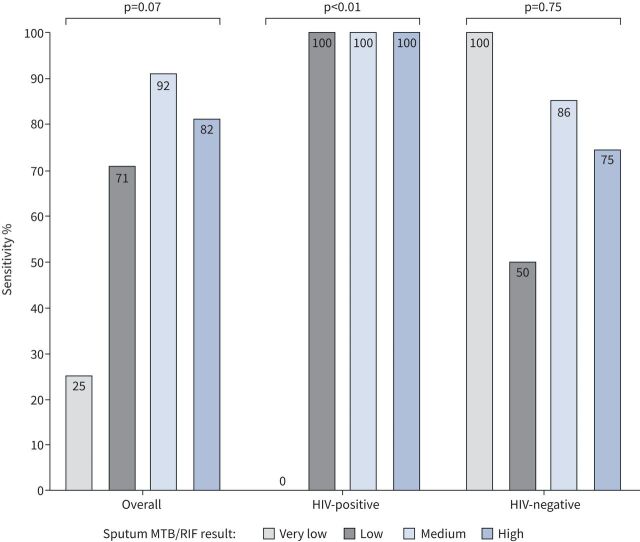
Association between FujiLAM sensitivity and Xpert MTB/RIF semiquantitative result according to HIV status among culture-confirmed tuberculosis cases (overall n=34 patients: HIV-positive n=12 patients and HIV-negative n=20 patients).

The relationship between FujiLAM positivity and Xpert MTB/RIF semiquantitative result among the 34 sputum culture-positive patients is shown in figure 4. Among HIV-positive patients, FujiLAM detected TB in all patients with low, medium or high sputum Xpert positive results, but missed TB in the three patients with a very low Xpert positive result, suggesting a relationship between FujiLAM and mycobacterial burden in HIV-positive patients. Among HIV-negative patients, there was no clear association between FujiLAM and the Xpert MTB/RIF semiquantitative result (figure 4).

## Discussion

In this first prospective study to evaluate the diagnostic accuracy of FujiLAM, we found that it had high sensitivity for the detection of TB among ambulatory adults with presumptive TB in Zambia. FujiLAM also demonstrated good specificity despite the lack of a comprehensive reference standard. Notably, FujiLAM's diagnostic accuracy did not substantially differ between HIV-positive and HIV-negative patients; however, our sample size was limited and additional, larger studies are needed to confirm these findings.

The diagnostic sensitivity of FujiLAM among HIV-positive patients in our study was consistent with prior evaluations. While our diagnostic accuracy results are informed by a relatively small number of ambulatory HIV-positive patients, it is encouraging that our results (sensitivity 75% (95% CI 43–95%)) are similar to previous evaluations conducted using biobanked specimens from three outpatient cohorts (pooled sensitivity 71% (95% CI 45–90%)) and three inpatient cohorts (pooled sensitivity 70% (95% CI 53–83%)) [7]. Among HIV-negative patients in our study, FujiiLAM's sensitivity (75% (95% CI 51–91%)) was higher than prior pooled estimates using biobanked specimens from three outpatient cohorts (53% (95% CI 44–62%)) [8]. While this may in part reflect the limited numbers of patients included and there may be no difference (overlapping confidence intervals), it may also reflect differences in the severity of TB disease and total mycobacterial burden among participants. FujiLAM's sensitivity appears to increase with greater mycobacterial burden [7, 8]; however, only 50% of HIV-negative patients in our study had smear-positive disease compared with 68% of patients in the previous study [8]. Differences in FujiLAM's sensitivity may also be due to differences in fresh *versus* frozen urine specimens; however, we previously found that only minor improvements in sensitivity may be observed when using fresh specimens [12]. Further studies will be needed to clarify this finding.

We found that the sensitivity of FujiLAM far exceeded that of smear microscopy (77% *versus* 44%). FujiLAM was able to detect TB in the large majority of those with smear-positive disease (87%) as well as the majority of those with smear-negative disease (64%). Our results suggest that FujiLAM used in conjunction with smear microscopy would nearly double the sensitivity of smear microscopy alone, but that smear microscopy would add little incremental yield beyond FujiLAM. Unfortunately, we were not able to directly compare FujiiLAM's sensitivity to the commercially available LF-LAM assay. However, in evaluations using biobanked specimens, FujiLAM had 35–40% greater pooled sensitivity among both HIV-positive (71% *versus* 35%) and HIV-negative patients (53% *versus* 11%) [7, 8].

FujiLAM's diagnostic sensitivity was less than that of Xpert MTB/RIF in this cohort, but comparable to that of Xpert Ultra among PLHIV with pulmonary TB in prior evaluations [13]. Roll-out of the Xpert platform in resource-constrained settings has faced challenges due to the need for a continuous power supply and the suboptimal laboratory conditions in primary healthcare settings that may result in prolonged down time [14]. Additionally, sputum Xpert may fail to detect TB among individuals with extrapulmonary and disseminated disease, a group in which FujiLAM appears to have high sensitivity but that we were unable to directly assess in the present study due to a lack of systematic extrapulmonary TB investigations [15]. Collectively, this suggests that FujiLAM could potentially be used as a first-line test to facilitate rapid TB diagnosis among presumptive TB patients, independent of HIV status, either as part of a parallel testing strategy with Xpert or in settings were Xpert remains unavailable.

Overall, FujiLAM had a good specificity (92%) that was consistent with previously published assessments (93%) [7, 8]. Reasons for potentially false-positive results need to be further explored in subsequent studies to clearly define patient populations among whom the test should be used to achieve sufficiently high specificity and avoid overtreatment. In the present study we did not systematically include extrapulmonary sampling to inform a comprehensive microbiological reference standard; this likely resulted in some misclassification, especially among HIV-positive patients as was suggested by the slightly lower specificity and PPV in this group compared with HIV-negative patients. For example, prior evaluations of FujiLAM using biobanked specimens have found its specificity to be up to 96% and 99% among HIV-positive and HIV-negative patients, respectively, when a composite reference standard including clinical diagnoses is utilised. Additionally, cross-reactivity with nontuberculous mycobacteria may have contributed to FujiLAM's reduced specificity in our study and should be specifically evaluated in future studies; however, preliminary evidence suggests that this is less problematic than for LF-LAM [16].

In conclusion, while larger prospective studies are required and are currently underway with the FujiLAM Prospective Evaluation Trial (ClinicalTrials.gov: NCT04089423), our results extend upon prior evaluations using biobanked specimens and suggest that the FujiLAM point-of-care test may have high sensitivity for the detection of TB among both HIV-positive and HIV-negative adults.

## Supplementary material

10.1183/13993003.03999-2020.Supp1 **Please note:** supplementary material is not edited by the Editorial Office, and is uploaded as it has been supplied by the author.
Supplementary tables ERJ-03999-2020.Supplement

## Shareable PDF

10.1183/13993003.03999-2020.Shareable1 This one-page PDF can be shared freely online.
Shareable PDF ERJ-03999-2020.Shareable
