# Supplementary material for: Diagnostic accuracy of a novel point-of-care urine lipoarabinomannan assay for the detection of tuberculosis among adult outpatients in Zambia: a prospective cross-sectional study
Source: Eur Respir J. 2021 Nov 18;58(5):2003999. doi: 10.1183/13993003.03999-2020 (PMC8631000; doi:10.1183/13993003.03999-2020)
Supplement: Supplementary file 1 [file ERJ-03999-2020.Supplement.pdf]

Supplementary Table S1. Diagnostic sensitivity and specificity of microbiological investigations for TB according to HIV-status among presumptive TB patients (n=151)

	**All patients (n=151)***				**HIV-positive (n=68)**				**HIV-negative (n=81)**			
	**n/N**	**Sensitivity (95%CI)**	**n/N**	**Specificity (95% CI)**	**n/N**	**Sensitivity (95%CI)**	**n/N**	**Specificity (95% CI)**	**n/N**	**Sensitivity (95%CI)**	**n/N**	**Specificity (95% CI)**
**Sputum smear microscopy**	15/34	44 (27-62)	117/117	100 (97-100)	3/12	25 (5-57)	56/56	100 (94-100)	10/20	50 (27-73)	61/61	100 (94-100)
**Sputum Xpert Ultra**	34/34	100 (90-100)	114/117	97 (93-100)	12/12	100 (74-100)	54/56	96 (88-100)	20/20	100 (83-100)	60/61	98.4 (91-100)
**Urine FujiLAM**	26/34	77 (59-89)	108/117	92 (86-96)	9/12	75 (43-95)	50/56	89 (78-96)	15/20	75 (51-91)	58/61	95 (86-99)
FujiLAM in Sp. Smear pos. (n=15)	13/15	87 (60-98)	-/-	-	3/3	100 (29-100)	-/-	-	8/10	80 (44-97)	-/-	-
FujiLAM in Sp. Smear neg. (n=138)	13/19	68 (43-87)	108/117	92 (86-96)	6/9	67 (30-93)	50/56	89 (78-96)	7/10	70 (35-93)	58/51	95 (86-99)

* 2 patients did not have a HIV-status results available.

Supplementary Table S2. Overview of patients who had a positive urine FujiLAM test but a negative sputum culture result (n=9)

Patient	Age	Sex	HIV-status	Prior history of TB	TB symptoms	Smear microscopy	Sputum Xpert result
1.	33	M	Neg	No	Cough x2 weeks, weight loss, night sweats, hemoptysis	Neg	Neg
2.	37	M	Neg	No	Cough x2 weeks, fever	Neg	Neg
3.	24	M	Neg	No	Cough x2 weeks, weight loss	Neg	Neg
4.	46	F	Pos	No	Cough x3 weeks, fever, night sweats	Neg	Neg
5.	36	F	Pos	Yes	Weight loss	Neg	Neg
6.	21	F	Pos	No	Cough x2 weeks, night sweats	Neg	Neg
7.	36	F	Pos	No	Cough x1 week, weight loss	Neg	Neg
8.	32	M	Pos	Yes	Cough x8 weeks, fever, weight loss, night sweats	Neg	Pos (low)
9.	48	M	Pos	Yes	Cough x7 weeks, fever, weight loss, night sweats	Neg	Neg

Abbreviations: F=female; M=male; Neg= negative; Pos=positive. Patient number 9 had evidence of non-tuberculous mycobacteria on sputum culture, however, this does not exclude the possibility of dual infection with *Mycobacterium tuberculosis*

Supplementary Table S3. Positive and negative predictive values of microbiological investigations for TB according to HIV-status among presumptive TB patients (n=151)

	All patients (n=151)				HIV-positive (n=68)				HIV-negative (n=81)			
	**n/N**	**PPV (95%CI)**	**n/N**	**NPV (95% CI)**	**n/N**	**PPV (95%CI)**	**n/N**	**NPV (95% CI)**	**n/N**	**PPV (95%CI)**	**n/N**	**NPV (95% CI)**
**Sputum smear microscopy**	15/34	100 (78-100)	117/117	86 (79-91)	3/12	100 (29-100)	56/56	86 (75-94)	10/20	100 (69-100)	61/61	86 (76-93)
**Sputum Xpert Ultra**	34/34	92 (78-98)	114/117	100 (97-100)	12/12	86 (57-98)	54/56	100 (94-100)	20/20	95 (76-100)	60/61	100 (95-100)
**Urine FujiLAM**	26/34	74 (57-88)	108/117	93 (87-97)	9/12	60 (32-84)	50/56	94 (84-99)	15/20	83 (59-96)	58/61	92 (82-97)
