# Supplementary material for: Diagnostic accuracy of a novel point-of-care urine lipoarabinomannan assay for the detection of tuberculosis among adult outpatients in Zambia: a prospective cross-sectional study
Source: Eur Respir J. 2021 Nov 18;58(5):2003999. doi: 10.1183/13993003.03999-2020 (PMC8631000; doi:10.1183/13993003.03999-2020)
Supplement: Supplementary file 2 [file ERJ-03999-2020.Shareable.pdf]

EUROPEAN RESPIRATORY JOURNAL
ORIGINAL RESEARCH ARTICLE
M. MUYOYETA ET AL.

SHAREABLE PDF

Diagnostic accuracy of a novel point-of-care urine lipoarabinomannan assay for the detection of tuberculosis among adult outpatients in Zambia: a prospective cross-sectional study

Monde Muyoyeta [1,4], Andrew D. Kerkhoff [2,4], Lophina Chilukutu[1], Emmanuel Moreau[3], Samuel G. Schumacher [3] and Morten Ruhwald[3]

[1]Centre for Infectious Diseases Research in Zambia, Lusaka, Zambia. [2]Division of HIV, Infectious Diseases and Global Medicine, Zuckerberg San Francisco General Hospital and Trauma Center, University of California San Francisco, San Francisco, CA, USA. [3]Foundation for Innovative New Diagnostics (FIND), Geneva, Switzerland. [4]These authors contributed equally to this work.

Corresponding author: Monde Muyoyeta (Mondemuy@gmail.com)

Check for updates

Shareable abstract (@ERSpublications)
In a prospective study, the FujiLAM urine-based point-of-care test demonstrated high sensitivity of ⩾75% for the detection of TB among both HIV-positive and HIV-negative ambulatory adults, and also had good specificity. https://bit.ly/328jr0d

Cite this article as: Muyoyeta M, Kerkhoff AD, Chilukutu L, *et al*. Diagnostic accuracy of a novel point-of-care urine lipoarabinomannan assay for the detection of tuberculosis among adult outpatients in Zambia: a prospective cross-sectional study. *Eur Respir J* 2021; 58: 2003999 [DOI: 10.1183/13993003.03999-2020].

This single-page version can be shared freely online.

Copyright ©The authors 2021.

This version is distributed under the terms of the Creative Commons Attribution Licence 4.0.

This article has supplementary material available from erj.ersjournals.com

Received: 29 Oct 2020
Accepted: 5 April 2021

Abstract

Background A novel, rapid, point-of-care urine-based lipoarabinomannan assay (Fujifilm SILVAMP TB LAM ("FujiLAM")) has previously demonstrated substantially higher sensitivity for tuberculosis (TB) compared with the commercially available Determine TB LAM assay using biobanked specimens. However, FujiLAM has not been prospectively evaluated using fresh urine specimens. Therefore, we determined the diagnostic accuracy of FujiLAM among HIV-positive and HIV-negative outpatients with presumptive TB in Zambia.

Methods Adult (⩾18 years old) presumptive TB patients presenting to two outpatient public health facilities in Lusaka were included. All patients submitted sputa samples for smear microscopy, Xpert MTB/RIF and mycobacterial culture, and urine samples for the FujiLAM assay. Microbiologically confirmed TB was defined by the detection of *Mycobacterium tuberculosis* in sputum using culture; this served as the reference standard to assess the diagnostic accuracy of FujiLAM.

Results 151 adults with paired sputum microbiological tests and urine FujiLAM results were included; 45% were HIV-positive. Overall, 34 out of 151 (23%) patients had culture-confirmed pulmonary TB. The overall sensitivity and specificity of FujiLAM was 77% (95% CI 59–89%) and 92% (95% CI 86–96%), respectively. FujiLAM's sensitivity among HIV-positive patients was 75% (95% CI 43–95%) compared with 75% (95% CI 51–91%) among HIV-negative patients. The sensitivity of FujiLAM in patients with smear-positive, confirmed pulmonary TB was 87% (95% CI 60–98%) compared with 68% (95% CI 43–87%) among patients with smear-negative, confirmed pulmonary TB.

Conclusions FujiLAM demonstrated high sensitivity for the detection of TB among both HIV-positive and HIV-negative adults, and also demonstrated good specificity despite the lack of systematic extrapulmonary sampling to inform a comprehensive microbiological reference standard.
